# SWITCHtoHEALTHY AI-Based Family Nutrition Recommendation System: Promoting the Mediterranean Diet

**DOI:** 10.3390/nu17243892

**Published:** 2025-12-12

**Authors:** Kyriakos Kalpakoglou, Perla Degli Innocenti, Federica Bergamo, Davide Beretta, Federico Bergenti, Alice Rosi, Francesca Scazzina, Lorena Calderón-Pérez, Noemi Boqué, Metin Güldaş, Çağla Erdoğan Demir, Lazaros P. Gymnopoulos, Kosmas Dimitropoulos

**Affiliations:** 1 Visual Computing Laboratory (VCL), Information Technologies Institute (ITI), Centre for Research and Technology Hellas (CERTH), 570 01 Thessaloniki, Greece; 2 Human Nutrition Unit, Department of Food and Drug, University of Parma, 43121 Parma, Italy; 3 Artificial Intelligence Laboratory, Department of Engineering for Industrial Systems and Technologies, University of Parma, 43121 Parma, Italy; 4 Technological Unit of Nutrition and Health, Eurecat, Technology Centre of Catalonia, 43204 Reus, Spain; 5 Department of Nutrition and Dietetics, Faculty of Health Sciences, Bursa Uludag University, 16059 Bursa, Türkiye; 6 Department of Biotechnology, Graduate School of Natural and Applied Sciences, Bursa Uludag University, 16059 Bursa, Türkiye

**Keywords:** AI-based meal recommender, AI-based nutrition recommender, Mediterranean diet, family meals, family diet, children meal recommender, healthy diet, artificial intelligence

## Abstract

**Background/Objectives:** Modern families face challenges in maintaining healthy and sustainable diets due to time constraints and busy lifestyles. The Mediterranean diet (MD), known for its benefits to both personal health and environmental sustainability, is often difficult to apply consistently within households. This paper presents and validates the SWITCHtoHEALTHY AI-based Family Nutrition Recommendation System, designed to generate meal plans aligned with MD guidelines. **Methods:** Two complementary recommendation engines were developed: the AI-based Family Nutritional Recommender, which creates personalized meal plans for adults that include shared family meals, and the Child Nutritional Recommender, which generates meal plans for children that could also incorporate school menus or proposals from the school cafeteria. Both systems rely on an expert-validated dataset of Mediterranean foods and are designed to comply with the expert-validated nutritional rules based on MD principals and national dietary guidelines. **Results:** The recommendation systems were validated using data from a real-world family intervention, achieving 90% accuracy in generating meal plans for all family members, while meeting the expert validated dietary rules for both adults and children. Moreover, AI-based Family Nutritional Recommender exceeds 90% accuracy in estimating calorie and nutrient content for adults. **Conclusions:** The results demonstrate the preliminary potential of AI-based recommendation systems to facilitate healthier and more sustainable dietary habits within modern households by generating personalized, nutritionally balanced family meal plans consistent with MD principles.

## 1. Introduction

Adopting a healthy and sustainable diet in today’s fast-paced world remains a significant challenge for families. Modern family lifestyles, often characterized by time constraints, competing responsibilities and mismatched schedules, frequently lead to reliance on convenience foods and unbalanced, improvised meals, all of which hinder consistent adherence to healthy eating habits [1]. At the same time, concerns about public health and environmental sustainability are growing, highlighting the urgent need for solutions that promote both individual well-being and sustainable food practices [2,3]. The MD has long been recognized as one of the healthiest and most sustainable dietary patterns, associated with reduced risk of chronic diseases, improved nutritional balance, and lower environmental impact [4,5]. Despite the growing awareness, individuals struggle to follow its principles even within the Mediterranean basin [6], and the process becomes even more complex at the family level [7]. Coordinating meals across children and adults, balancing school-provided menus with home-prepared meals and accommodating diverse preferences and restrictions all add layers of difficulty.

In recent years, growing attention has been given to the development of nutrition recommender systems, mobile applications, and digital tools aimed at supporting healthier eating behaviors through personalized guidance [8,9]. Users increasingly embrace these technologies, reporting that they help change dietary behavior, improve diet quality, and mitigate risk factors [10,11], with health experts also beginning to adopt and recommend them in patients [12]. These technologies make use of advances in Artificial Intelligence (AI) to interpret user information, such as dietary habits, lifestyle factors, and cultural preferences, and translate it into tailored nutritional advice. Broadly, nutritional recommendation systems can be categorized into traditional approaches, combinatorial optimization methods, knowledge-based algorithms, and more recently, generative AI and large language model (LLM) approaches [13,14].

Traditional approaches employ strategies such as content-based filtering, collaborative filtering, or hybrid approaches. Content-based filtering recommends food items by matching them to the attributes and preferences recorded in a user’s profile, or by suggesting items similar to those the user has previously selected [15,16]. In contrast, collaborative filtering generates recommendations by comparing users to one another: it identifies individuals with similar behavior or preferences and proposes items that these like-minded users have rated or consumed [17,18]. Hybrid techniques combine both strategies, integrating profile-based similarity with user-to-user correlations, in order to deliver recommendations that are more accurate, diverse, and resilient to the limitations of either method on its own [19,20]. Despite their usefulness, traditional recommendation approaches face key limitations, including data sparsity, the cold-start problem, and limited capacity to address complex user needs or incorporate nutritional guidelines.

Combinatorial optimization methods, such as the knapsack algorithm, integer programming, or linear programming, synthesize meal plans by selecting the best combinations of food items considering nutritional adequacy, user preferences, and cost [21,22,23,24]. More advanced many-objective optimization (MaOO) approaches [25] explicitly balance nutritional values, dietary diversity, and user preferences, providing a more holistic framework for meal recommendations. Mechanisms such as the Meal Plan Generator (MPG) [26] synthesize meals from foods while respecting caloric intake and variety.

Knowledge-based AI nutritional recommenders [27,28,29] incorporate multiple factors (e.g., nutritional needs, personal preferences, dietary guidelines, seasonality, locality, food group diversity) to generate weekly meal plans. These systems are grounded in validated nutritional knowledge, which ensures that recommendations are both safe and aligned with established dietary standards; they achieve high accuracy in recommendations but rely heavily on expert-validated guidelines. ML models further extend knowledge-based capabilities by learning patterns from large datasets, enabling highly personalized daily meal plans that adapt to evolving user behaviors [30]. Most recently, generative AI models, particularly LLMs such as ChatGPT, have emerged as flexible tools capable of producing personalized meal plans [31,32], offering a wide range of recommendations that combine nutritional goals with contextual adaptability. However, machine learning and generative AI models carry the risk of hallucinations and generating unrealistic or incorrect suggestions.

Despite the growing number of nutritional recommendation systems and digital tools, the vast majority are designed for individuals rather than households. Existing approaches typically generate meal plans tailored to a single user’s profile, overlooking the unique challenges of family-based recommendations. At the family level, meal planning must reconcile diverse nutritional needs, preferences, age-specific requirements and dietary restrictions, while also considering practical constraints such as time, mismatching schedules, and school or workplace meals. Moreover, it must optimize for shared meals among family members, which introduces an additional layer of combinatorial complexity that individual-focused systems are not designed to handle. To date, existing recommender systems have rarely been designed to comprehensively address these complexities, highlighting a critical gap in AI-driven approaches to family nutrition.

To address these challenges, the SWITCHtoHEALTHY (S2H) AI-based family nutrition recommendation system (AIFNRS) is proposed, integrating the AI-based family Nutritional recommender (AIFNR) and the Child Nutritional Recommender (CNR), which work in tandem to offer healthy meal proposals to all family members. The CNR builds weekly Nutritional Plans (NPs) tailored to children (3–11 years old) by also considering their school menus or proposals from the school cafeteria and expert-validated nutritional rules. On the other hand, the AIFNR generates weekly NPs, including shared family meal proposals, by integrating user’s nutritional needs and preferences, expert-validated nutritional guidelines, diversity and food group variety. For both systems we chose a knowledge-based approach, grounded in expert-validated nutritional rules, as the superior and safer choice for family and child meal planning—where shared meals and school menus must be incorporated into the recommenders, and where ensuring safety and high nutritional accuracy is paramount. Moreover, systems adhere to MD principles [27] and draw on an expert-validated dataset of Mediterranean meals and dishes curated for this purpose. Finally, a dedicated web application was implemented to enable families to track their tailored meal proposals.

Overall, the main contributions of this work are summarized as follows: First, we propose the AIFNRS, which delivers healthy, balanced, and personalized weekly meal plans for all family members, following expert-validated nutritional rules and adhering to MD principles. Second, we introduce the S2H family Mediterranean meal and dish dataset, which has been enhanced with expert-validated Mediterranean meals and dishes as well as children’s school menus. Finally, we validate the AIFNR and CNR systems using real family profiles, demonstrating their effectiveness in generating personalized and nutritionally adequate family meal plans.

## 2. Materials and Methods

### 2.1. The SWITCHtoHEALTHY Family Mediterranean Meal and Dish Dataset

The S2H family Mediterranean meal and dish dataset [33] was developed to supply the recommendation systems with curated Mediterranean foods. Specifically, the AIFNR utilizes meals and dishes from the S2H dataset to generate personalized meal plans for adults, while, the CNR employs the same dataset to produce weekly meal suggestions for children, ensuring that both home-cooked menus and those in accordance with the school menus or the offerings from the school cafeteria are balanced. The structure and content of the dishes, meals and school menus that populate the dataset is presented below.

*Dish: unique identification (ID); name or short description; ingredients (for adult and child age groups); recipe; tip; nutritional information (calories, fat, protein, carbohydrates); dish type (*e.g.,* semi, unique); food group included in the dish (*e.g.,* processed meat, legumes, fish); and color of vegetables (*e.g.,* red, green).*

Dishes can range from single items such as “*banana*” to more complex preparations such as “*Scrambled eggs with prawns and wild asparagus*”. For each dish, detailed ingredient information is provided (differing for adults and the various children’s and adolescents’ age groups, from 3 to 17 years old) along with the recipe preparation procedure and the related educational content as a short tip (about its nutritional, culinary, preservation and sustainability aspects). In addition, nutritional composition (calories, fat, protein, and carbohydrates) is specified just for adults. This information was not considered necessary for children, as nutrition education on consumption frequency and food portions was prioritized, providing plans with standard portions for each age group, according to the national dietary guidelines and MD principles. Finally, each dish is associated with one or more food groups, covering a total of 26 food group categories (see Section 2.4.1 for more details). In Table 1, two examples of Spanish dishes are presented.

*Meal: unique identification (ID); name or short description; type (breakfast, lunch, dinner, morning and afternoon snacks); country (Spain, Türkiye); season (summer, spring, winter, autumn); and associated dish/dishes (from one to up to ten).*

Simple meals include only one dish, such as “*Porridge with seasonal fruits*”, while more complex meals combine several dishes, such as “*Tangerine, banana and milk milkshake with whole-wheat bread and olive oil*”, which consists of five dishes. Meals are categorized into five types: breakfast, lunch, dinner, and morning and afternoon snack. Each meal is associated with one of the two countries covered in the project (Spain and Türkiye), ensuring cultural and dietary relevance. Additionally, information on the preferred season of consumption (winter, spring, summer, autumn) is provided for each meal to align with seasonality and the principles of the MD. In Table 2, two representative meal examples are presented.

School menus and proposals from the school cafeteria:

School menus consist of five school meals (type of lunch), corresponding to the lunches offered to children (3 to 11 years old) at school from Monday to Friday. Following the same structure used for meals and dishes, school meals are associated with school dishes, with the following differences:

- *School dish: Nutritional information (calories, fat, protein, carbohydrates) is not included.*
- *School meal: Additional information is provided, including the date and the name of the school where the lunch was served.*

Proposals from the school cafeteria consist of the same information described for dishes and meals from school menus, apart from the date on which the meal is provided. These proposals are always available during the school week; thus, it is not required to provide a specific reference day for each one.

#### Dataset Construction

The S2H Family Mediterranean meal and dish dataset consists of Mediterranean foods curated by the expert team of nutritionists in each research center (Spain, Türkiye) and verified by nutritionists from the S2H consortium. The dataset was populated to provide complete weekly menus for all family members (adults, children and adolescents), covering five daily meal options (breakfast, lunch, dinner and both morning and afternoon snacks). In total, the dataset consists of 262 meals, emphasizing nutrient-rich ingredients, such as fruits, vegetables, legumes, whole grains, and healthy fats (particularly olive oil), while limiting processed foods and added sugars. Additionally, local ingredients and healthy cooking methods were also prioritized to ensure both authenticity and sustainability. For the Spanish dataset, dietary guidelines from the Public Health Agency of Catalonia [34] were taken into account. For the Turkish database, the guidelines of the Turkish Ministry of Health, the Turkish Ministry of Agriculture and Forestry, and the 2022 Türkiye Nutrition Guide published by the General Directorate of Public Health of Türkiye [35] were considered. Table 3 outlines the fundamental principles used for implementing meals and dishes in both countries.

Food items from the dataset were designed to cater to all members of a family. Consequently, portion sizes were adjusted according to the age and nutritional requirements of each individual, ranging from adults to different child and adolescent age groups. In Table 4, standard portion sizes used for the curation of Spanish dishes across different age groups are presented.

**Figure 1:**
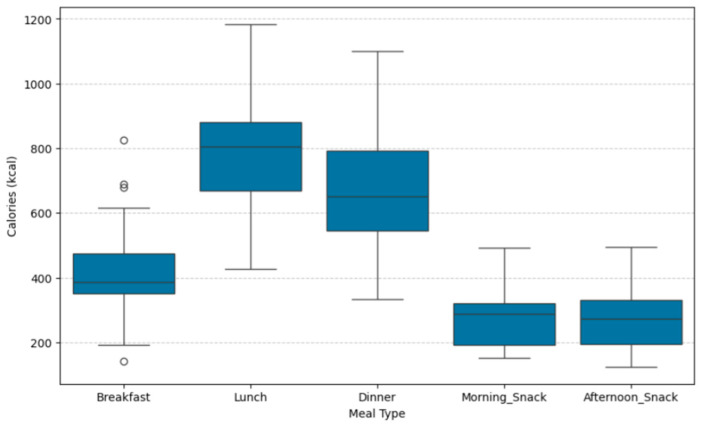
Boxplot of calorie distribution (kcal/meal) across Spanish meal type categories. Boxes show the interquartile range (IQR), with horizontal lines indicating the median calorie content. Whiskers extend to the minimum and maximum values within 1.5 × IQR; outliers are displayed as individual points.

**Figure 2:**
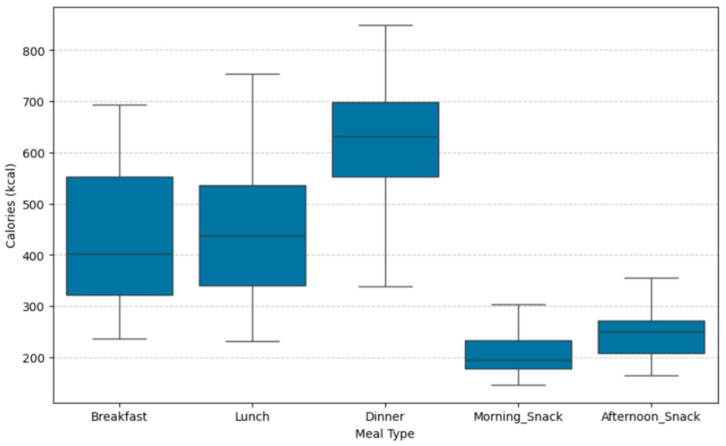
Boxplot of calorie distribution (kcal/meal) across Turkish meal type categories. Boxes show the interquartile range (IQR), with horizontal lines indicating the median calorie content. Whiskers extend to the minimum and maximum values within 1.5 × IQR; outliers are displayed as individual points.

The S2H Family Mediterranean meal and dish dataset represents an extended version of the Mediterranean meal and dish dataset presented thoroughly in [27]. The initial dataset included a diverse selection of Spanish and Turkish meals curated by expert nutritionists in accordance with the principles of the MD. However, this dataset exhibited some limitations, which were addressed through targeted enhancements. (i) The caloric ranges of the generated NPs—composed of breakfast, morning snack, afternoon snack, lunch and dinner—did not fully cover the needs of users with either low or high energy requirements in Spain, nor those with moderate to high energy requirements in Türkiye. To address this issue, new meals and dishes were added to the dataset, and existing ones were modified to cover a broader energy spectrum. (ii) The initial dataset had limited capacity to accommodate users with specific allergies. More specifically, for users with egg and fish allergies the available options were limited, whereas for nut and milk allergies, the options were nearly zero. To address these limitations, the dataset was expanded to provide a greater number of available options for users with these allergies. In particular, for milk allergy, the dataset was redesigned to include dairy-free alternatives. (iii) To enhance nutritional diversity and improve food group tracking, new food group labels were introduced, including categories such as processed meat, chicken, turkey, rabbit, chickpeas, lentils, white/red beans, and other legumes. These enhancements allow the recommendation engines to generate more varied and personalized weekly NPs. In total, 82 new meals were added to the dataset, and several existing ones were updated or refined, resulting in a final dataset of 283 meals and 464 dishes. Figure 1 and Figure 2 show the caloric distribution of the 186 Spanish meals and 97 Turkish meals, categorized by meal type.

Additionally, school menus were included into the database to have the information needed about the lunches actually consumed by children (3–11 years old) at school. In total, 494 menus (401 dishes; 728 meals) were collected in Spain and 11 menus (88 dishes; 110 meals) in Türkiye. The wide variation in the number of school menus reflects the number of participating schools (40 in Spain and 1 in Türkiye), which resulted in different school proposals being available. Additionally, one extra menu (7 dishes; 7 meals) was collected in Türkiye from a school cafeteria, due to the availability of this service.

For the purposes of the S2H project, in addition to English, each dataset was also translated into the local country’s language.

### 2.2. Child Nutritional Recommender (CNR)

The CNR was devised to provide healthy and balanced weekly Mediterranean NPs:

- For children (3–11 years old) having lunch at school: Based on the lunches offered in the school menu (Monday to Friday), the system generates proposals for all other meals during the week (breakfasts, snacks, dinners, and weekend meals).
- For children (3–11 years old) eating at the school cafeteria: According to the lunches offered at the school cafeteria (Monday to Friday), the system provides proposals for all other meals throughout the week (breakfasts, snacks, dinners, and weekend meals). Unlike school menus, proposals from the school cafeteria are not specific to each day of the week. The service offers similar meals each day (e.g., “*cheddar toast and kefir*”, “*cheese pancake and milk*”), allowing flexibility in the order of lunch proposals during the week.
- For children (3–11 years old) with meals prepared at home: When school menus are not available, the system independently generates weekly meal plans without constraints imposed by school or cafeteria menus.

Although the CNR was primarily designed for younger children (3–11 years), it can also be applied to older siblings (adolescents, 12–17 years) by generating the same meal plans offered to their younger sibling with portions appropriate for their age group. This is because the adolescents aged 12–17 years enrolled in the project usually did not eat at school or no school meal service was available. Therefore, it was not possible to follow the same procedure used for children aged 3–11 years. However, to ensure that families could have similar menus for all members, while adapting them to the specific needs of each age group, this option was chosen within the CNR functionalities.

Several rules were defined to balance nutritional adequacy, feasibility, and variety, combining the MD principles with national dietary guidelines for children summarized in Table 3. Within the CNR, rules were structured at both the weekly and daily level, specifying the allowed frequencies for different food groups and subgroups within the generated menus. The CNR generates weekly plans using meals, dishes and school menus selected from the S2H family Mediterranean meal and dish dataset (Section 2.1).

At the weekly level, the rules define both minimum and maximum frequencies for the main food categories. Among the weekly rules, pulses are recommended between 4 and 5 times per week. Fish and seafood are scheduled to appear 2 to 4 times per week. White meat is limited to a maximum of 2 servings per week, while the combined intake of red and processed meat cannot exceed 2 portions in total. Carbohydrates have a recommended frequency of 21 to 35 servings per week, distributed among bread, pasta, rice, tubers, and cereals. Finally, fruit and vegetables are strongly promoted, with 21 to 28 weekly portions of fruit and 14 to 21 portions of vegetables. At the daily level, rules ensure balanced distribution. Pulses are limited to 2 portions per day, milk and yogurt to 3 portions, cheese to 1 portion, and eggs to 1 portion. Carbohydrates are limited to 3 portions of bread, 1 portion of pasta and one portion of rice, tubers or cereals per day. Furthermore, fruit (≥3 portions) and vegetables (≥2 portions) are encouraged daily, ensuring adequate intake of plant-based foods. Additional color-based rules are also implemented to guarantee variety in the intake of vegetables. Since weekly menus are also available for children with milk protein allergies, the same rules are applied; however, meals containing milk or dairy products are excluded and replaced with those featuring suitable dairy substitutes.

Notwithstanding, if school menus are found to be non-compliant with the established rules, they cannot be altered and are instead accepted by the CNR system. For instance, if the school menu exceeds the weekly limit for red meat (e.g., two servings instead of the maximum of one), the recommender automatically avoids proposing red meat in the remaining meals of that week. For the intervention groups in which the consumption of S2H snacks is foreseen, these are provided 3 times per week, either as a mid-morning or an afternoon snack. Finally, for children with milk allergy, dairy products are systematically excluded from the menu and replaced with plant-based alternatives, to preserve both nutritional adequacy and dietary safety. For the dairy-free menus, all weekly and daily rules are applied but those specifically related to dairy products are not considered, as no products containing milk could be used by the system and are replaced by dairy-free alternatives. The constraint is set to zero for lactose-containing foods (for milk and yogurt and for cheese) and the same minimum and maximum daily and weekly limits are applied to the corresponding dairy-free alternative groups (plant-based beverages and yogurt, and plant-based cheese alternatives). Substitutions of dairy products with dairy-free alternatives occurred mainly for breakfasts and snacks, when milk and yogurt are replaced by soy milk/yogurt or with other plant-based beverages, to maintain variety and acceptability.

With numerous constraints to consider, e.g., school menus to complement and a plethora of nutritional rules, the system applies a set of main rules to be preferred and a complementary set of less restrictive rules. These alternative rules are not intended to replace the stricter criteria in their entirety, but rather to serve as flexible adjustments when the system is unable to provide a feasible menu. Typically, only one or two rules are relaxed at a time, depending on the limiting food category in the school-provided menus. Finally, weekly limits have been set for both meal and dish IDs to prevent over-repetition of the proposals. Table 5 presents the expert-validated nutritional rules used by the CNR.

From a technical perspective, the CNR is formalized as a constraint satisfaction problem (CSP) and follows a four-step process to provide weekly meal plans for children. Those steps are presented below.

- **Step 1: Specify Recommender Inputs**. Six input parameters are provided to the system: *season, country, school, date range of the week, milk allergy*, and *snack preference*. The parameters *country*, *school*, and *date range of the week* are used to retrieve the corresponding school menu, while the remaining ones guide the selection of the additional meals in the generated menu. Specifically, *season* and *snack preference* determine the meal options available for the given context, and the *milk allergy* parameter ensures that only dairy-free meals are included when required.
- **Step 2: Check for School Menus.** Based on the *country*, *school*, and *date range of the week* inputs, the system checks whether a school menu exists. If a menu is available, school menus are retrieved from the dataset and used as predefined weekday lunches by the CNR in step 3. When a school menu is not specified for a child, the recommender selects lunches from the S2H family Mediterranean meal and dish dataset. Finally, the recommender treats the school cafeteria menus like a school menu, but the lunches are not associated with specific days; the order in which the system selects cafeteria lunches depends on the search strategy used.
- **Step 3: Child Menu Generation.** The best possible weekly plan is generated based on the input data, the school or school cafeteria menu (if provided) and the expert-validated nutritional rules presented in Table 5. When a school or school cafeteria menu is available, the system uses the predefined school lunches (from Monday to Friday) and generates the remaining meals accordingly. Specifically, the CNR automatically counts how many daily and weekly portions of each food category (listed in Table 5) are already present in the school or school cafeteria menu. It then compares these values with the daily and weekly consumption frequencies defined by the expert-validated nutritional rules. Based on the portions that can still potentially be included for each food category, the CNR generates the NPs. If no school or school cafeteria menu is provided, the system generates all meals independently. If no valid solution can be found for a given week, the search strategy is adjusted or the constraints are relaxed to allow the generation of an acceptable menu. The output is a list of meal identifiers representing each day of the week.
- **Step 4: Data Storing and Sharing.** The generated menus are uploaded to a database, ensuring they can be retrieved at any time. The weekly menus are made available to other applications via a web API. Through the same API, the AIFNR retrieves the necessary meals to generate weekly menus tailored for the adults in the family.

The CNR has been initially developed as an independent tool. Therefore, aside from the actual recommender, a web application has been developed to show the generated menus to the users. The web application served as a showcase for the CNR, and it is an interactive tool that allows the user to navigate the menus (previous, current and next week) for different children of different schools.

### 2.3. AI-Based Family Nutritional Recommender (AIFNR)

The AIFNR was designed to provide healthy, balanced and personalized weekly Family NPs to adults, considering the users’ nutritional needs and preferences, expert-validated nutrition guidelines, the principles of the MD, diversity and food group variety rules and finally the children’s meal suggestions generated from the CNR. The AIFNR generates weekly plans using meals and dishes selected from the S2H family Mediterranean meal and dish dataset (Section 2.1).

The AIFNR was developed on top of the core framework of the AI-based Adult Nutritional Recommender (AIANR) presented in [27]. Adaptations were introduced to extend the system from an individual-based approach to a family-oriented one. The adapted AIFNR workflow is presented below.

- **Step 1: Meal Filtering.** Meals are first filtered based on the user’s preferred cuisine and any declared allergies (Section 2.3.1).
- **Step 2: Children’s Meal Suggestions.** Seven daily dinners and two weekend lunches generated by the CNR are provided as inputs for the AIFNR (Section 2.3.2).
- **Step 3a: Create Daily Meal Combinations.** For each day, all possible combinations of the remaining meals (after filtering in step 1) are generated in conjunction with the predefined children’s meals. These combinations form seven lists of potential daily meal plans (Section 2.3.3).
- **Step 3b: Sort Daily Plans.** Each list of daily meal plans is sorted from most to least suitable based on how closely each plan aligns with the user’s nutritional needs and macronutrient intake (Section 2.3.3).
- **Step 4: Build the Weekly Plan**. The best possible plan from each daily list is selected to create a full weekly plan, ensuring it follows the MD principles alongside balanced food group intake and meal/dish variety throughout the day and week (Section 2.3.4).

#### 2.3.1. Meal Filtering

Meals are filtered based on user allergy (milk protein) and cuisine preferences (Spanish, Turkish) primarily to protect users’ health and, secondarily, to support a more sustainable and culturally relevant diet. Allergies are considered a critical factor, as they help prevent potentially harmful reactions. For instance, milk allergy is among the most common in both children and adults and can trigger a range of symptoms, from gastrointestinal symptoms (e.g., diarrhea, abdominal pain, nausea) to severe reactions like anaphylaxis [36]. Cuisine preference, in contrast, supports personalization and promotes the consumption of familiar, culturally appropriate foods. Prioritizing local cuisine not only enhances user satisfaction but also encourages the use of seasonal and locally sourced ingredients, contributing to environmental sustainability and supporting local food systems [37].

#### 2.3.2. Children’s Meal Suggestions

To generate family meal proposals, the AIFNR uses predefined meals provided by the CNR: seven daily dinners and two weekend lunches. These meals serve as fixed inputs for the AIFNR, which then explores all possible combinations using the remaining available meals from the S2H Family Mediterranean meal and dish dataset.

#### 2.3.3. Creation and Sorting of Daily Family NPs

For each day of the week, the algorithm generates a list of 100,000 randomly created meal combinations. On weekdays, each daily plan includes a randomly selected breakfast, morning snack, lunch, and afternoon snack, paired with the fixed dinner. On weekends, the predefined lunch and dinner are combined with random selections of breakfasts, morning snacks, and afternoon snacks. Each list is then scored and ranked from most to least optimal using the same process employed by the AIANR, as described in detail in [27]. Briefly, to score and rank the daily NPs, the recommender first calculates user-specific parameters, including Age, Body Mass Index (BMI), Basal Metabolic Rate (BMR), and Daily Energy Requirements (DER). Specifically, DER is derived from BMI, BMR, and Physical Activity Level. Each daily NP is then assigned a Daily Nutritional Plan Score (DNPS), defined as the sum of four partial scores: Caloric Score (CS), Protein Score (PS), Fat Score (FS), and Fruit and Vegetable Score (FVS). The CS measures how closely the plan’s total calories match the user’s DER, while the PS and FS evaluate whether protein and fat contributions fall within expert-validated nutritional guidelines (15–20% of DER for protein, 25–40% of DER for fat). The FVS measures whether the plan includes between 5 and 10 fruit and vegetable servings per day, in line with expert guidelines. In this way, the system ensures that proposed plans are nutritionally balanced, realistic, sustainable, and health-promoting. Finally, based on the DNPS, daily NPs are sorted from the most to least optimal.

#### 2.3.4. Building the Weekly Family Nutritional Plan (FNP)

Diversity and MD rules are key factors in ensuring that users receive a wide range of meals and dishes with varied food groups, in alignment with the principles of the MD, one of the healthiest and most extensively studied dietary patterns. These rules help prevent monotony in meal plans, promote nutritional balance, and support long-term adherence to healthy eating habits. The AIFNR iteratively examines the daily NPs within each of the seven daily lists until it identifies one daily NP from each list that, together, forms a complete weekly plan. These selected daily NPs are chosen to ensure that the overall weekly plan meets the diversity, food group variety, and MD guidelines, outlined in Table 6.

### 2.4. Technical Implementation

#### 2.4.1. Application Database

The S2H database consists of twelve tables, each carefully structured to support both the user experience and the meal-planning process. Of these, six tables are dedicated to user-related information, four tables store the S2H family Mediterranean meal and dish dataset information, and the remaining two tables are designed to store the personalized meal plans recommended to users.

User related Tables: The “*User*” table serves as the primary entry point, storing account credentials. From this table, each account can register a family by creating up to two adult user profiles and up to eight child profiles. The adult “*UserProfile*” table stores the key information required by the Adult Family Nutritional Recommender (AFNR) to generate personalized meal plans, including sex, year of birth, height, weight, physical activity level, dairy allergy, and country. Similarly, the “*ChildProfile*” table contains the data necessary for tracking school menus and generating child-specific meal suggestions (CNR), with fields such as school name, age group, milk allergy, snack preferences, school lunch participation, and country. The remaining three tables (“UserProfileHistory”, “trackUserActions”, and “trackUserProfileActions”) are dedicated to monitoring changes in user profiles and logging user interactions within the application. These logs capture actions such as logins and button clicks, allowing the system to track usage patterns and levels of user engagement.

S2H Family Mediterranean Meal and Dish Tables: The “Meal” and “Dish” tables represent a central component of the S2H database, storing the school menus and the collection of the Mediterranean meals and dishes. In the “*Meal*” table, each entry is associated with as many as ten dishes, referenced through foreign keys to the “*Dish*” table. The “Meal” table also classifies meals according to their type (breakfast, morning snack, lunch, afternoon snack, or dinner) and include an indicator specifying whether the meal is appropriate for adult or child recommendations, since only meal for adults contain nutritional information (calories, fat, protein, carbohydrates). The “*Country*” field further assigns each meal to its respective cuisine, ensuring alignment with local dietary contexts, and the “*Season*” field specifies the season (Winter, Spring, Summer, Autumn) for which a meal is appropriate.

The “*Dish*” table stores the essential nutritional composition of each dish, including total calories, fat, protein, and carbohydrates. It also incorporates a set of Boolean fields that specifies the presence or absence of particular food components, such as processed meat, white meat, chicken, turkey, rabbit, red meat, pork, fish, pulses, chickpeas, lentils, white/red beans, other legumes, dairy products, dairy substitutes, eggs, pasta, rice, tubers, soups, cereals, fruits, nuts, raw vegetables, and cooked vegetables. When vegetables are included in a dish, their colors are specified. In addition, for each dish, the table records the list of ingredients with their respective quantities (adapted for adults and different child and adolescent age groups), the recipe preparation and a short educational tip.

Finally, the “*MealLanguage*” and “*DishLanguage*” tables store translations of meal and dish information, ensuring accessibility for users in multiple languages. For the purposes of the S2H project, the content was translated into English, Spanish and Turkish.

Nutritional Meal Planning-related Tables: The “*NP*” and “*NPMeal*” tables are used to store and track the weekly meal plans generated for each adult user. Daily NPs are stored in the “*NP*” table, with one entry per day, specifying both the day of the week (e.g., Monday, Tuesday, etc.) and also the date range of the corresponding week (e.g., 7–13 September 2025). For each NP, five associated “*NPMeal*” entries are created to store the individual daily meals: breakfast, morning snack, lunch, afternoon snack, and dinner.

#### 2.4.2. The Web Application

Although the focus of this paper is on the presentation of the family nutrition recommendation system, for the sake of completeness, this section also describes the web application developed to enable families to access and follow their personalized meal plan recommendations. Upon accessing the application, users are navigated to the login page. After authentication—or registration, if no account has been created—the users are navigated to the “*User Profiles*” screen, where all family profiles are displayed. An ‘Add’ button, located next to the last profile, allows the creation of additional profiles (adult or child) by completing the required form fields. By selecting their profile, adult users are able to view the weekly meal plans generated by the AFNR for the current and previous weeks, along with detailed information on meals and dishes, including nutritional values, ingredients, recipes, and tips. The “*Charts*” section provides analytics on caloric intake, nutrient distribution, and food group categories at both weekly and daily levels. Two additional sections, “*Educational Materials*” and “*Game*”, support user engagement and learning. Users are also able to update their personal information via the “*User Profile*” section. By selecting a child profile, users see the child meal plans generated by the CNR. In contrast to adult profiles, child profiles do not have access to the “*Educational Materials*” or “*Charts*” sections. A detailed site map of the S2H web application is presented in Supplementary Materials. The main application screens are subsequently presented.

Login/Register: On the registration page, users are required to provide a username, password, and email address. The email address is essential, as a verification message is automatically sent to confirm the account. For security purposes, users have to create a strong password that meets predefined requirements. Once registration is completed and the account is successfully activated through email verification, the user is able to log in and access the application.

User Profiles: On the “*User Profiles*” page, the user can view all profiles that have been created for the family, whether adult or child. Each profile provides direct access to its corresponding home page. An “*Add*” button on this page allows the creation of a new adult (up to two) or child profile (up to eight) by completing the required input form. The necessary input fields—many of which are essential for the AIFNR and CNR—are detailed in Table 7.

**Figure 3:**
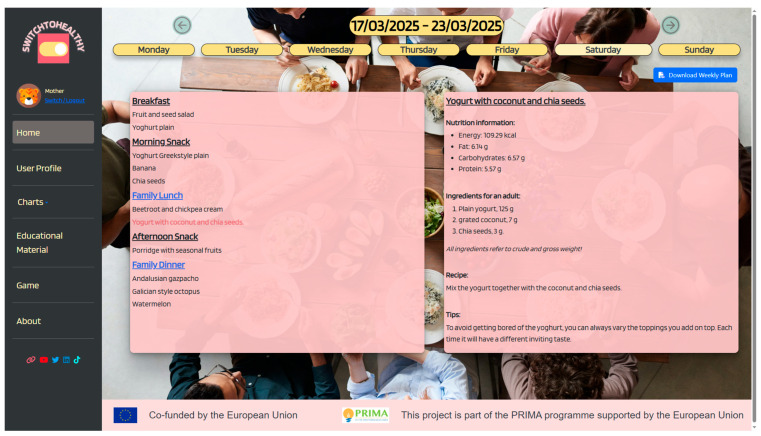
Home screen of the S2H web application.

Home: From the home screen, both adult and child users can access their personalized weekly meal plans. Navigation is intuitive, allowing users to first select the desired week and then the specific day. For each selected day, the complete daily meal plan is displayed, with the option of browsing individual meals. Users can explore each meal in detail, including nutritional information (calories, fat, protein, carbohydrates), ingredient lists, preparation instructions (recipes), and tips to support home preparation. Meals that appear in both adult and child plans are clearly highlighted and labeled as “*family meals*”, enabling users to easily identify shared eating occasions. Figure 3 illustrates the home screen of the S2H web application.

Charts: Visual diagrams illustrate weekly energy intake, macronutrient distribution, and food group consumption, providing valuable insights into how well the recommended plans align with individual nutritional requirements and dietary guidelines. This functionality is available exclusively for adult profiles and is intentionally omitted from child profiles.

Educational Materials: Within the web application, educational contents were specifically developed for adult participants of the PRIMA S2H project [39], tackling several themes related to the MD principles and practical aspects to manage a healthy and sustainable diet. The purpose of these materials is to increase parents’ nutritional knowledge and thus improve their skills in managing their children’s diets. Accordingly, 17 topics are structured: *Welcome Topic—The meaning of a diet and healthy eating; Topic 1—Mediterranean Diet; Topic 2—Lifestyle; Topic 3—Healthy and sustainable diet; Topic 4—Nutrients; Topic 5—Food groups; Topic 6—Food seasonality; Topic 7—Drinks and hydration; Topic 8—Meals; Topic 9—The “healthy eating plate”; Topic 10—The breakfast; Topic 11—Food consumption frequencies; Topic 12—Food portions; Topic 13—Learning from mistakes!; Topic 14—Recognizing hunger; Topic 15—Eating out; Topic 16—Grocery shopping and cooking.* All topics are accessible through the app, each accompanied by infographics that can be downloaded in PDF format. These contents have been developed by a team of nutritionists involved in the PRIMA S2H project based on several scientific references [40,41,42,43] and adapted to each country when specific recommendations apply.

Educational Game: Within the framework of the PRIMA S2H project, we designed and developed a “Tamagotchi”-like game for mobile devices [44]. By combining engaging gameplay with educational content, the S2H game promotes healthier dietary habits through the MD principles. In addition to its other functionalities, the game teaches players about balanced and varied diets and food groups, by focusing on reducing poor dietary intake, and encouraging physical activity. All family members are able to engage with the game to foster healthier eating behaviors and lifestyle habits.

## 3. Experimental Results

This section presents the validation of the AIFNR and CNR systems, focusing on their ability to generate appropriate weekly meal plans, aligned with MD principles, for all family members. The validation was conducted using real family profiles collected from participants in the S2H intervention study. This study is a parallel, randomized, single-blinded, and controlled multicentric nutritional intervention designed to validate the efficacy of a multi-component intervention to promote a sustainable shift toward the MD pattern within families. Conducted in Spain and Türkiye, the study aimed to involve 320 families with adolescents and children to evaluate the effects of a multi-component intervention combining digital interactive tools (S2H web application), hands-on educational materials and activities, and healthy, sustainable plant-based snacks. By using a full-factorial design, families were randomized into eight groups (one control and seven interventions) to test the independent and combined effects of each component (S2H web application and/or educational materials and/or snacks), while also examining the influence of socioeconomic, dietary, lifestyle, and environmental factors on family eating behaviors. More details about the S2H intervention study are presented in the S2H study protocol [38].

From the families participating in the study, our focus for the presentation of the experimental results is on the intervention groups that interacted with the S2H web application. Within this framework, 42 families participated in the app-using groups, and valid usage data were obtained from 32 of them. Across these families, 57 adult profiles and 61 child profiles were created. These families formed the analysis sample for validating the performance of both the CNR and AIFNR systems, as well as the AIFNRS.

For this work, the real profiles created with the S2H web application were used only for the validation of the two recommendation systems. The presentation of the intervention, as well as any behavioral outcomes associated with it, are beyond the scope of this paper.

### 3.1. The Child Nutritional Recommender (CNR) Validation

The validation results for the NPs generated and delivered through the CNR are presented as follows. Specifically, daily and weekly compliance with the expert-validated nutritional rules (Table 5) is described in Table 8 and Table 9, respectively. These results group data from all the NPs provided to real family profiles. The NPs were based on the school menus or the school cafeteria’s proposals that were effectively eaten by children (see Section 2.1), or on meals to be prepared at home when the former were not consumed.

As shown in Table 8, overall daily NPs (*n* = 7406) exhibit a high level of compliance across all rules. For the 19 preferred rules, the mean compliance was 96%. When the 9 less restrictive rules (related to rice, tubers, cereals, fruit, and vegetable color groups) were applied together with the 10 unchanged rules, mean compliance increased to 99%.

Full compliance (100%) was achieved for purple vegetables (≤2 portions/day), making the corresponding less restrictive rule (≤3 portions/day) unnecessary. Among the eight items where less restrictive rules were applied, the greatest improvements were observed for tubers (+12%) and cereals (+31%), which originally showed the lowest adherence under the preferred rules (≤1 portion/day for both).

With the adjusted thresholds, compliance for green and yellow vegetables reached 100% (≤3 portions/day for both). For all items with unchanged rules, compliance remained consistently high (≥97%).

With respect to the weekly rules, results are presented in Table 9. Weekly NPs showed moderate adherence when applying the 23 preferred rules, with a mean compliance of 71%. When switching to the 12 less restrictive rules (covering red and processed meat, fish/seafood, pulses—including chickpeas, lentils, white/red beans, and other pulses—cheese, rice, tubers, cereals, and vegetables) together with the unchanged rules, compliance improved to 91%.

Full compliance with the preferred rules was observed for rabbit (≤1 portion/week), pasta (≤7 portions/week), rice (≤7 portions/week), and bread (≤14 portions/week). In contrast, preferred rules were never met for white/red beans and other pulses in Türkiye: the former consistently exceeded the maximum limit (≤1 portion/week) while the latter was not offered at all in the menu.

The adoption of less restrictive rules resulted in 100% compliance for fish/seafood (2–5 portions/week) and for tubers and cereals (≤14 portions/week). Substantial improvements were also seen in fish/seafood (+65%), pulses (+51%), white/red beans (+59%), other pulses (+78%), cheese (+64%), and vegetables (+53%). However, the limits for pulses were still exceeded in 6% (*n* = 438) of weekly NPs, and only 0.1% (*n* = 6) fell below the minimum intake of four portions. For cheese, 5% (*n* = 314) of NPs exceeded the maximum allowed intake (>4 portions/week).

Among the rules that remained unchanged, compliance was generally high (≥83%), except for fruit, which showed the lowest adherence (53%) relative to the recommended range (21–28 portions/week). In addition, milk and yogurt (14–21 portions/week) were below the lower threshold in 2% (*n* = 167) of Turkish NPs.

For fruit, 6% (*n* = 436) exceeded the upper weekly limit, while 1% (*n* = 65) fell below the minimum. Almost all menus below the requirement (98.5%, *n* = 65) originated from Türkiye; however, these menus still provided at least 15 portions per week.

Throughout the intervention, flexibility in both daily and weekly dietary rules was allowed to support realistic compliance while still promoting balanced nutrition. Certain rules, such as daily diversity in colors of vegetables or weekly subcategories of carbohydrates and pulses, could be excluded without compromising overall nutritional quality (as mentioned in Table 5). For the remaining components, the system was adapted case-by-case by adjusting or relaxing requirements to ensure that all NPs remained healthy and balanced.

Additionally, a more detailed breakdown of the NPs proposed in Spain and Türkiye is available in the Supplementary Materials. These data are classified into NPs generated based on school menus, school cafeteria proposals or meals prepared at home.

### 3.2. AI-Based Family Nutritional Recommender (AIFNR) Validation

The AIFNR was evaluated with 57 adult profiles. Across these profiles, the system generated 476 daily NPs, of which 434 successfully passed the diversity, food group variety and MD rules presented in Table 6. Forty-two plans did not meet these criteria, since children’s meal suggestions already violate them.

More specifically, the AIFNR achieved high accuracy in meeting nutritional requirements across calories, macronutrients (fat and protein), and fruit and vegetable intake for adults. Overall, the algorithm reached more than 90% accuracy in calories, over 80% accuracy in macronutrients, and 100% accuracy in fruits and vegetables. Comprehensive statistics from the AIFNR, both overall and separated by country (Spain and Türkiye), are presented in Table 10.

### 3.3. Summary

The integrated CNR and AIFNR systems successfully generated personalized NPs for both children and adults across 32 real family profiles, producing 7406 daily NPs for children and 476 NPs for adults (434 of which were eligible for validation).

For children, compliance with daily nutritional rules was very high. The system achieved an average adherence of 96% for the 19 preferred rules, which further increased to 99% when the 9 less restrictive rules and 10 unchanged rules were applied. Regarding weekly nutritional rules, compliance reached 71% under the preferred criteria and improved to 91% with the less restrictive approach.

For adults, 434 of the generated NPs met the diversity, food group variety, and Mediterranean diet rules. Accuracy in meeting key nutritional requirements was high: more than 90% for calories, above 80% for macronutrients (fat and protein), and 100% for fruit and vegetable intake. Only 42 plans did not meet all criteria due to incompatibilities introduced by children’s meal constraints.

Overall, these results demonstrate that the AIFNRS achieves 90% accuracy (434 out of the 476 NPs) and effectively delivers healthy, personalized, nutritionally balanced and MD-compliant meal plans for families, while successfully integrating shared meals and school/cafeteria-based menus.

## 4. Discussion

Overall, the AIFNR and CNR achieve high accuracy in proposing NPs for all family members, including shared meals. This approach could therefore support parents in managing a healthy and balanced diet for their children and themselves, while also helping them meet their nutritional needs. The validation, conducted with 32 real family profiles, demonstrated the system’s effectiveness. Among daily family NPs generated, a high accuracy in proposing meals that complied with the expert-validated nutritional rules and the MD principles for both children and adults was achieved. For children, daily compliance was high irrespective of the rules applied (preferred or less restrictive) and so was the weekly adherence with the less restrictive criteria. Furthermore, for adults, the AIFNR achieved high accuracy in aligning with caloric targets, macronutrient distributions, and fruit and vegetable suggestions, further demonstrating the system’s capability to support users in meeting their individual caloric and macronutrient goals while following shared family meal suggestions.

Further improvements would enhance the system’s functionality. Firstly, incorporating more dishes and meals into the datasets would facilitate the AIFNRS in providing healthy and balanced NPs. This would result in an increased variety of combinations for daily and weekly meals, which could also benefit compliance with nutritional rules for children and adults. A larger dataset would also allow for diversification of the NPs over the weeks, possibly meeting the needs of families who prefer less monotonous diets. Moreover, as previously mentioned, country-specific datasets were used to develop the NPs in order to follow national dietary guidelines and culinary traditions as closely as possible. In some cases (e.g., cereals, pulses, fruits), this resulted in discrepancies between Spain and Türkiye in terms of compliance with the AIFNR and CNR standards. Better adherence to MD rules could be achieved by adapting these to each country’s context. Moreover, expanding the dataset would not only allow the app to be implemented in additional Mediterranean regions, but also support different culinary traditions, food preferences, and nutritional needs (e.g., food allergies) within the same country.

Nonetheless, certain limitations should be acknowledged. One limitation relates to the portion system: the gram values defining each portion may slightly deviate from standard reference portions, even though they are still counted as equivalent. This can result in less precise portion-based rules, potentially leading to underestimation or overestimation of specific food items within the menus.

Additionally, challenges may arise when NPs are generated by the CNR using school lunch menus as a base. In this study, for example, some menus failed to meet the weekly recommendations for meat intake because the upper limit for meat consumption had already been reached through school-provided meals. Therefore, close collaboration with school catering services is essential to ensure that baseline school menus used for NP generation are compatible with MD principles and aligned with national dietary guidelines. A similar issue may occur when shared meal proposals generated by the CNR do not satisfy the daily and weekly nutritional rules for adults used by the AIFNR. To address these challenges, a negotiation protocol should be developed between the AIFNR and CNR to resolve rule conflicts.

Moreover, the school menus and the proposals from the school cafeteria used by the CNR to provide the NPs were collected by the team of each research center. Consequently, implementing this system into a real-life context and everyday use could be challenging: the CNR and the whole AIFNRS should be implemented to gather information on school proposals from users, or trained personnel should support the functioning of the system.

Furthermore, the AIFNRS provides family meal plans derived from a single child’s menu: as described previously, once the NP is generated by the CNR, the AIFNR produces plans for adults based on it. The tool may thus struggle to incorporate this information and provide shared meal proposals for families featuring more children with different dietary needs (e.g., due to diverse school menus).

Overall, this study is among the first to develop and technically validate an AI-based family nutrition recommendation system capable of generating coordinated meal plans for adults and children, including shared meals, while adhering to expert-validated nutritional rules and MD principles. Validation using data from 32 real family profiles further supports the system’s preliminary potential and practical relevance. Nevertheless, several limitations should be acknowledged. Conflicts may arise when school lunch menus or shared meals do not align with nutritional rules, underscoring the need for closer collaboration with school catering services and the development of a negotiation protocol between the AIFNR and CNR. In addition, school menus were collected manually, suggesting the need for automated menu acquisition in real-life use. Finally, the current system generates family meal plans based on a single child’s NP, which limits its applicability to families with multiple children with differing dietary needs. Addressing these limitations in future work will enhance the system’s usability, scalability, and real-world implementation potential.

## 5. Conclusions

This study introduced AIFNRS, a recommendation system that supports the adoption of MD within households. By combining the CNR and the AIFNR, the system offers healthy, personalized and balanced weekly meal suggestions to families, ensuring they follow expert-validated nutritional guidelines, food group variety and the principles of the MD. The integration of an enriched Mediterranean meal and dish dataset further ensures that recommendations are nutritionally accurate, culturally relevant, and sustainable.

Validation with real family profiles demonstrated that the proposed system is capable of effectively generating meal plans for all family members, incorporating shared meal proposals and children’s school menus, while fostering adherence to MD principles and expert-validated nutritional rules. To explore the long-term adherence and the system’s impact on family dietary habits, a longitudinal study has been designed [38]. Future work will focus on expanding the meal and dish dataset, with particular emphasis on high-protein plant-based options to better meet macronutrient targets across all user groups. Moreover, we aim to develop a negotiation protocol between the AIFNR and CNR to resolve rule conflicts in shared meals. These improvements will help adapt the AIFNRS to real-life use and further enhance the role of AI-based tools in guiding families toward healthier and more sustainable dietary practices.

## Figures and Tables

**Table 1:** Examples of Spanish dishes retrieved from the SWITCHtoHEALTHY family Mediterranean meal and dish dataset.

ID	Dish Name	Ingredients (Standard Portion for Adults)	Nutritional Composition	Food Components	Recipe
4	Banana	Banana, 160 g	Kcal: 114.03; Protein: 2.13 g; Fat: 1.38 g; Carbs: 15.49 g	Fruit	Keep in mind that the weight of the fruit in this recipe is expressed as gross weight, with any skin or stone it may have. Don’t throw old fruit in the bin! Try to reduce food waste by making tasty smoothies or shakes
45	Scrambled eggs with prawns and wild asparagus	Egg, 65 g; Small peeled prawns, 50 g; Wild asparagus, 30 g; Spring garlic, 5 g; Salt, 1 g; Olive oil, 10 g	Kcal: 210.67; Protein: 14.6 g; Fat: 15.78 g; Carbs: 2.3 g	Eggs; Fish or seafood; Cooked vegetables	Boil the asparagus for 2 min. and drain. Beat the eggs with the salt. Heat a non-stick pan and add the oil. Brown the garlic and sauté the prawns for 2–3 min. over medium heat. Add the beaten eggs and lower the heat. Mix continuously until the egg is curdled.

**Table 2:** Examples of Spanish and Turkish meals retrieved from the SWITCHtoHEALTHY family Mediterranean meal and dish dataset.

ID	Meal Name	Type	Country	Season	Associated Dish ID (Name)
4	Porridge with seasonal fruits	Breakfast	Spain	Autumn; Winter; Spring; Summer	33 (Porridge with seasonal fruits)
336	Oatmeal with tomato pastry, arugula salad and lemonade	Lunch	Türkiye	Autumn; Spring; Summer	335 (Oatmeal with tomato pastry); 365 (Fresh Lemonade); 456 (Arugula salad)

**Table 3:** Key nutritional principles for meal and dish implementation according dietary guidelines of the Public Health Agency of Catalonia [34] and guidelines of the Turkish Ministry of Health, the Turkish Ministry of Agriculture and Forestry, and the 2022 Türkiye Nutrition Guide published by the General Directorate of Public Health of Türkiye [35].

Key Nutritional Principles for Meal and Dish Implementation		
	Spain	Türkiye
Carbohydrates, fibers and their main sources	o Encourage wholegrain versions of bread, pasta, and cereals at all main meals. o Sugars should be <10% of total energy intake; ideally <5%. For a 2000 kcal diet: 25–50 g/day. Children: max 25 g/day. o Minimum 5 servings/day of fruits and vegetables (3 fruits + 2 vegetables). Prioritize fresh, seasonal, and local produce.	o It is recommended that 45–60% of daily dietary energy comes from carbohydrates. o Complex carbohydrates with a low glycemic index should be preferred as carbohydrate sources. o At least 25 g of fiber should be consumed daily. o Foods high in fiber, such as fruits, vegetables, legumes, and wholegrain products, should be included in the daily diet. o At least 5 servings of vegetables and fruits should be consumed daily (at least 400 g/day). Vegetables and fruits should be chosen in season.
Proteins and their main sources	o At least 3–4 times per week in varied preparations (stews, salads, spreads, etc.). o Limit to max 1–2 servings of red meat per week. Favor white meats and plant-based proteins.	o It is recommended that 10–20% of daily energy comes from protein. o All age groups should consume milk and dairy products daily.
Fats and their main sources	o Use extra virgin olive oil as the main fat source (4–6 servings/day ≈ 10 mL each). o Avoid butter, margarine, and refined oils. o A daily handful of nuts is recommended (unsalted, unroasted). For children ≤ 3 years: ground or in paste.	o It is recommended that 20–35% of daily dietary energy should come from fat. o It is important to consume polyunsaturated fatty acids instead of saturated fats. o Consumption of foods containing trans fats should be reduced.
Sodium and its main sources	o Max 5 g/day (≈1 tsp) of salt, including hidden sources. Always iodized salt. o Avoid habituating children to salty flavors.	o Daily salt consumption should not exceed 6 g.
General principles	o Water is the preferred and recommended beverage at all times. Avoid sugary drinks, juices, and alcohol. o Ultra-processed foods are strongly discouraged. Choose fresh/minimally processed foods, particularly important for children’s diets. o Prioritize seasonal and locally produced foods to support sustainability and freshness.	o When planning a meal, it is important to ensure a balanced distribution of carbohydrates, proteins, fats, and vegetables. o Dietary diversity is the basis of a healthy diet. o Boiling, steaming, baking, and grilling should be preferred over frying.

**Table 4:** Portion sizes used for the curation of Spanish dishes across different age groups.

Food Group	Age Groups				
	3–6 Years	7–12 Years	13–15 Years	16–18 Years	Adults
Vegetables (g)	120–150	120–150	150–25	150–250	150–250
Meat, boneless (g)	40–50	60–80	90–110	90–110	100–120
Meat (with bone) (g)	55–70	80–110	120–150	120–150	140–160
Fish (g)	50–60	70–90	100–130	100–130	100–150
Eggs (g)	40	55–110	70–110	70–110	70–110
Legumes, main (g)	30–50	50–60	60–80	80–100	60–80
Legumes, side (g)	15–20	20–30	30–40	40–50	30–40
Potatoes, main (g)	100–150	100–150	150–200	200–250	150–200
Potatoes, side (g)	55–65	65–75	80–95	110–130	80–95
Rice/Pasta, main (g)	50–60	60–80	80–90	90–100	60–80
Rice/Pasta, side (g)	20–25	25–30	30–35	35–50	25–30
Bread, main (g)	65–75	80–100	100–115	115–130	80–100
Bread, side (g)	20–40	30–40	40–50	40–60	30–60
Oil (mL)	10	12	15	15	15

**Table 5:** Expert-validated nutritional rules used by the Child Nutritional Recommender (CNR).

Weekly Rules			
Repetition of a Meal’s ID at maximum 2 times/week; repetition of a Meal’s ID must not occur on consecutive days			
**Food Categories**	**Preferred Rules**	**Less Restrictive Rules**	**Notes**
**Pulses**	4 ≤ portions ≤ 5	4 ≤ portions ≤ 6	If the subclassifications of legumes are too restrictive, only the macro category of legumes can be considered.
White/red beans; Other pulses	1 portion	1 ≤ portions ≤ 2	
Lentils; Chickpeas	1 ≤ portions ≤ 2	1 ≤ portions ≤ 3	
**Dairy Products**	/	**/**	
Milk and Yogurt	14 ≤ portions ≤ 21	not to be changed	
Cheese	2 ≤ portions ≤ 3	2 ≤ portions ≤ 4	
Dairy-free Products *	/	**/**	
Plant-based beverages and Yogurt	14 ≤ portions ≤ 21	not to be changed	
Plant-based Cheese	2 ≤ portions ≤ 3	2 ≤ portions ≤ 4	
**Fish or Seafood**	2 ≤ portions ≤ 4	2 ≤ portions ≤ 5	
**White Meat**	≤2 portions	not to be changed	If the subclassifications of white meat are too restrictive, only the macro category of white meat can be considered.
Turkey; Rabbit	≤1 portion	not to be changed	
Chicken	≤2 portions	not to be changed	
**Red Meat**	≤1 portion	(Red meat + Processed meat) ≤ 2 portions	
**Processed Meat**	≤1 portion		
**Eggs**	1 ≤ portions ≤ 3	not to be changed	
**Carbohydrates**	21 ≤ portions ≤ 35	not to be changed	
Bread	≤14 portions	not to be changed	
Tubers; Rice; Cereals	≤7 portions	≤14 portions	
Pasta	≤7 portions	not to be changed	
**Fruit**	21 ≤ portions ≤ 28	not to be changed	
**Vegetables**	14 ≤ portions ≤ 21	14 ≤ portions ≤ 28	
**Daily Rules**			
**Food Categories**	**Preferred Rules**	**Less Restrictive Rules**	**Notes**
**Pulses**	≤ 2 portions	not to be changed	
White/red beans; Other pulses; Lentils; Chickpeas	**/**	**/**	
**Dairy Products**	**/**	**/**	
Milk and Yogurt	≤3 portions	not to be changed	
Cheese	≤1 portion	not to be changed	
Dairy-free Products *	/	/	
Plant-based beverages and Yogurt	≤3 portions	not to be changed	
Plant-based Cheese	≤1 portion	not to be changed	
**Fish or Seafood**	≤1 portion	not to be changed	
**White Meat**	(White meat + red meat + processed meat) ≤ 1 portion	not to be changed	
Turkey; Chicken; Rabbit			
**Red Meat**			
**Processed Meat**			
**Eggs**	≤1 portion	not to be changed	
**Carbohydrates**	**/**		
Bread	≤3 portions	not to be changed	
Tubers; Rice; Cereals	≤1 portion	≤2 portions	
Pasta	≤1 portion	not to be changed	
**Fruit**	≥3 portions	not to be changed	
**Vegetables**	≥2 portions	not to be changed	
**Colors of Vegetables**	/	/	If the colors are too restrictive, do not include them in the rules.
Red; Green; White; Yellow; Purple; Multicolor	≤2 portions	≤3 portions	

* Dairy-free product rules are used only for users with milk allergy, instead of those for dairy products.

**Table 6:** Diversity, food group variety and the MD rules for the Adult AI-based Family Nutritional Recommender (AIFNR).

Mediterranean Diet Rules	
Rule	Foods Concerned
≤1 time/day	Eggs
≤1 time/week	Turkey; Rabbit
≤2 times/week	Red meat; Chicken; White/red beans; Other legumes; Processed meat
≤3 times/week	Chickpeas; Lentils; Rice; Pasta
≤4 times/week	Red and white meat ^1^
≤6 times/week	Pulses; Fish or seafood ^2^
**Diversity and Food Group Variety Rules**	
Rule	Foods Concerned
≤1 time/day	Fruit salad; Repeating dishes
≤1 time/day in lunch or dinner	White meat; Red meat; Pork; Fish; Pasta
≤2 times/week	Fruit salad; Repeating meals

^1^ Red and white meat together can be consumed up to four times per week. ^2^ A maximum of three times per week as main dish and four times per week as side dish.

**Table 7:** Input fields for adult and child profiles.

Adults Form		
	Field	Explanation
Personal Information	Username	
	Profile Image	
Physical characteristics	Sex [Male, Female]; Year of Birth; Height, in m; Weight, in kg	Essential inputs for BMI and BMR calculations.
Physical Activity Level	Sedentary (=1.2); Lightly active (=1.375); Moderately active (=1.55); Very active (=1.725); Extra active (=1.9)	Essential input to estimate Daily Energy Requirements (DER).
Allergies	Milk Protein	Indicates whether the user has a milk protein allergy.
Country	Spain; Türkiye	Defines the local cuisine from which the user will receive meal proposals.
Plans presented in	Selected country language; English	Sets the primary language that meals will be presented.
Interface Language	English; Spanish; Turkish; French	Sets the primary language for the user interface.
Intervention Questions	Did you receive the project snacks for the children?	Two intervention questions for classifying users to the four intervention groups, enrolled into the healthy snacks or educational materials use [38].
	Did you receive the educational materials and activities for the adolescents?	
**Children Form**		
Personal Information	Username	
	Profile Image	
School Related Information	School Name	The child’s school, used for tracking school-provided menus.
	School Lunch	Specifies whether the child participates in school lunches, to provide or not weekly menus based on the school menu or on the proposals from the school cafeteria.
Physical characteristics	Group Age	The child’s age classification (3–6, 7–12, 13–15, 16–18), for providing the correct ingredient portions (see Table 4).
Allergies	Milk Protein	Indicates whether the child has a milk protein allergy.
Country	Spain; Türkiye	Defines the local cuisine from which the child will receive meal proposals.
Plans presented in	Selected country language; English	Sets the primary language that meals will be presented.
Interface Language	English; Spanish; Turkish; French	Sets the primary language for the user interface.
Intervention	SwitchtoHealthy Snack	Specifies whether the child receives the project healthy snack [38].

**Table 8:** Compliance of daily NPs with the daily rules from the CNR, displayed for Spain, Türkiye and overall.

Compliance of Daily NPs with the Daily Rules from the CNR									
Food Items	Spain (*n* = 6944)			Türkiye (*n* = 462)			Overall (*n* = 7406)		
	Preferred Rule *n* (%)	Less Restrictive Rule *n* (%)	Delta %	Preferred Rule *n* (%)	Less Restrictive Rule *n* (%)	Delta %	Preferred Rule *n* (%)	Less Restrictive Rule *n* (%)	Delta %
Pulses	6802 (97.96)	Not to be changed		462 (100.00)	Not to be changed		7264 (98.08)	Not to be changed	
Milk and Yogurt	6810 (99.68) ^2^	Not to be changed		462 (100.00)	Not to be changed		7272 (99.70) ^2^	Not to be changed	
Cheese	6712 (98.24) ^2^	Not to be changed		458 (99.13)	Not to be changed		7170 (98.30) ^2^	Not to be changed	
Plant-based beverages and Yogurt ^3^	112 (100.00)	Not to be changed					112 (100.00)	Not to be changed	
Plant-based Cheese ^3^	110 (98.21)	Not to be changed					110 (98.21)	Not to be changed	
Fish or seafood	6909 (99.50)	Not to be changed		462 (100.00)	Not to be changed		7371 (99.53)	Not to be changed	
All meat ^1^	6832 (98.39)	Not to be changed		462 (100.00)	Not to be changed		7294 (98.49)	Not to be changed	
Eggs	6861 (98.80)	Not to be changed		462 (100.00)	Not to be changed		7323 (98.88)	Not to be changed	
Bread	6942 (99.97)	Not to be changed		461 (99.78)	Not to be changed		7403 (99.96)	Not to be changed	
Tubers	6082 (87.59)	6935 (99.87)	12.28	430 (93.07)	462 (100.00)	6.93	6512 (87.93)	7397 (99.88)	11.95
Rice	6654 (95.82)	6943 (99.99)	4.16	462 (100.00)			7116 (96.08)	7405 (99.99)	3.90
Pasta	6936 (99.88)	Not to be changed		462 (100.00)	Not to be changed		7398 (99.89)	Not to be changed	
Cereals	4870 (70.13)	6937 (99.90)	29.77	211 (45.67)	461 (99.78)	54.11	5081 (68.61)	7398 (99.89)	31.29
Fruit	6933 (99.84)	Not to be changed		265 (57.36)	Not to be changed		7198 (97.19)	Not to be changed	
Vegetables	6944 (100.00)	Not to be changed		454 (98.27)	Not to be changed		7398 (99.89)	Not to be changed	
Red vegetables	6853 (98.69)	6938 (99.91)	1.22	457 (98.92)	462 (100.00)	1.08	7310 (98.70)	7400 (99.92)	1.22
Green vegetables	6737 (97.02)	6944 (100.00)	2.98	434 (93.94)	462 (100.00)	6.06	7171 (96.83)	7406 (100.00)	3.17
White vegetables	6901 (99.38)	6944 (100.00)	0.62	431 (93.29)	458 (99.13)	5.84	7332 (99.00)	7402 (99.95)	0.95
Yellow vegetables	6919 (99.64)	6944 (100.00)	0.36	462 (100.00)			7381 (99.66)	7406 (100.00)	0.34
Purple vegetables	6944 (100.00)			462 (100.00)			7406 (100.00)		
Multicolor vegetables	6381 (91.89)	6929 (99.78)	7.89	460 (99.57)	462 (100.00)	0.43	6841 (92.37)	7391 (99.80)	7.43

Data are presented as numbers of daily NPs (%). ^1^ All meat includes processed, red and white meat. ^2^ Data on milk and yogurt and on cheese does not include NPs for users with milk protein allergy in Spain (*n* = 112 daily NPs), as the related food was not provided. The rules for all other food items were the same for both the regular menu and those for children with milk protein allergy. ^3^ The rules for dairy-free products refer just to NPs for users with milk protein allergy in Spain (*n* = 112 daily NPs).

**Table 9:** Compliance of weekly NPs with the weekly rules from the CNR, displayed for Spain, Türkiye and overall.

Compliance of Weekly NPs with the Weekly Rules from the CNR									
Food Items	Spain (*n* = 992)			Türkiye (*n* = 66)			Overall (*n* = 1058)		
	Preferred Rule *n* (%)	Less Restrictive Rule *n* (%)	Delta %	Preferred Rule *n* (%)	Less Restrictive Rule *n* (%)	Delta %	Preferred Rule *n* (%)	Less Restrictive Rule *n* (%)	Delta %
Pulses	21 (2.12)	554 (55.85)	53.73	56 (84.85)	60 (90.91)	6.06	77 (7.28)	614 (58.03)	50.76
Chickpeas	752 (75.81)	942 (94.96)	19.15	34 (51.52)	34 (51.52)	0.00	786 (74.29)	976 (92.25)	17.96
Lentils	866 (87.30)	965 (97.28)	9.98	60 (90.91)	60 (90.91)	0.00	926 (87.52)	1025 (96.88)	9.36
White/red beans	393 (39.62)	966 (97.38)	57.76	0 (0.00)	54 (81.82)	81.82	393 (37.15)	1020 (96.41)	59.26
Other pulses	112 (11.29)	935 (94.25)	82.96	0 (0.00)	0 (0.00)	0.00	112 (10.59)	935 (88.37)	77.79
Milk and yogurt	880 (100.00) ^1^	Not to be changed		11 (16.67)	Not to be changed		891 (85.51) ^1^	Not to be changed	
Cheese	23 (2.61) ^1^	662 (75.23) ^1^	64.42 ^1^	14 (21.21)	46 (69.70)	48.48	37 (3.55) ^1^	708 (67.95) ^1^	64.40 ^1^
Plant-based beverages and Yogurt ^2^	15 (93.75)	Not to be changed					15 (93.75)	Not to be changed	
Plant-based Cheese ^2^	3 (18.75)	14 (87.50)	68.75				3 (18.75)	14 (87.50)	68.75
Fish or seafood	308 (31.05)	992 (100.00)	68.95	66 (100.00)			374 (35.35)	1058 (100.00)	64.65
Processed meat Red meat	733 (73.89)	955 (96.27) ^3^		66 (100.00)	66 (100.00) ^3^		799 (75.52)	1021 (96.50) ^3^	
	884 (89.11)			43 (65.15)			927 (87.62)		
White meat	931 (93.85)	Not to be changed		66 (100.00)	Not to be changed		997 (94.23)	Not to be changed	
Chicken	958 (96.57)	Not to be changed		66 (100.00)	Not to be changed		1024 (96.79)	Not to be changed	
Turkey	990 (99.80)	Not to be changed		66 (100.00)	Not to be changed		1056 (99.81)	Not to be changed	
Rabbit	992 (100.00)	Not to be changed		66 (100.00)	Not to be changed		1058 (100.00)	Not to be changed	
Eggs	815 (82.16)	Not to be changed		64 (96.97)	Not to be changed		879 (83.08)	Not to be changed	
Carbohydrates	985 (99.29)	Not to be changed		64 (96.97)	Not to be changed		1049 (99.15)	Not to be changed	
Bread	992 (100.00)	Not to be changed		66 (100.00)	Not to be changed		1058 (100.00)	Not to be changed	
Tubers	988 (99.60)	992 (100.00)	0.40	66 (100.00)			1054 (99.62)	1058 (100.00)	0.38
Rice	992 (100.00)			66 (100.00)			1058 (100.00)		
Pasta	992 (100.00)	Not to be changed		66 (100.00)	Not to be changed		1058 (100.00)	Not to be changed	
Cereals	843 (84.98)	992 (100.00)	15.02	6 (9.09)	66 (100.00)	90.91	849 (80.25)	1058 (100.00)	19.75
Fruit	556 (56.05)	Not to be changed		1 (1.52)	Not to be changed		557 (52.65)	Not to be changed	
Vegetable	427 (43.04)	985 (99.29)	56.25	66 (100.00)			493 (46.60)	1051 (99.34)	52.74

Data are presented as numbers of weekly NPs (%). ^1^ Data on milk and yogurt and on cheese does not include NPs for users with milk protein allergy in Spain (*n* = 16 weekly NPs), as the related food was not provided. The rules for all other food items were the same for both the regular menu and those for children with milk protein allergy. ^2^ The rules for dairy-free products refers just to NPs for users with milk protein allergy in Spain (*n* = 16 weekly NPs). ^3^ The less restrictive rule applies to processed meat along with red meat.

**Table 10:** Caloric, macronutrient (fat, protein) and fruit and vegetable accuracy for Spanish and Turkish adult users.

	Overall Statistics of the AIFNR					
	Weekly NPs	Daily NPs	Mean Caloric Agreement	Fat Within Range	Protein Within Range	Fruit and Veg Within Range
Spain	46	322	95.16%	80.95%	90.16%	100%
Türkiye	16	112	89.38%	87.75%	68.37%	100%
Overall	59	434	93.80%	82.57%	84.99%	100%

## Data Availability

The original data presented in the study are openly available in SWITCHtoHEALTHY Mediterranean Meals and Dishes Dataset, e.g., Zenodo at https://doi.org/10.5281/zenodo.17514151 (accessed on 8 September 2025).

## Supplementary Materials

The following supporting information can be downloaded at: https://www.mdpi.com/article/10.3390/nu17243892/s1, Table S1: Compliance of daily NPs in Spain and Türkiye with the daily rules from the CNR, categorized into those based on school menus, school cafeteria proposals or meals prepared at home; Table S2: Compliance of weekly NPs in Spain and Türkiye with the daily rules from the CNR, categorized into those based on school menus, school cafeteria proposals or meals prepared at home; Figure S1: Site map of the SWITCHtoHEALTHY Family Application.

## Abbreviations

MD	Mediterranean diet
S2H	SWITCHtoHEALTHY
AIFNRS	AI-based family nutrition recommendation system
AIFNR	AI-based family nutritional recommender
AIINR	AI-based adult nutritional recommender
CNR	Child nutritional recommender
NPs	Nutritional plans
AI	Artificial intelligence
ML	Machine learning
LLMs	Large language models
ChatGPT	Chat Generative Pre-trained Transformer
BMI	Body mass index
BMR	Basal metabolic rate
DER	Daily energy requirements
PAL	Physical activity level
DNPS	Daily nutritional plan score
CS	Caloric score
PS	Protein score
FS	Fat score
FVS	Fruit and vegetable score
