# Supplementary material for: SWITCHtoHEALTHY AI-Based Family Nutrition Recommendation System: Promoting the Mediterranean Diet
Source: Nutrients. 2025 Dec 12;17(24):3892. doi: 10.3390/nu17243892 (PMC12735497; doi:10.3390/nu17243892)

***Supplementary Table S1**. Compliance of daily NPs in Spain and Türkiye with the daily rules from the CNR, categorized into those based on school menus, school cafeteria proposals or meals prepared at home.*

COMPLIANCE OF DAILY MENUS WITH THE DAILY RULES FROM THE CNR															
	Spain (n=6944)						Türkiye (n=462)								
	NPs based on school menu (n=6804)			NPs for meals prepared at home (n=140)			NPs based on school menu (n=154)			NPs based on school cafeteria (n=154)			NPs for meals prepared at home (n=154)		
Food items	Preferred rule n (%)	Less restrictive rule n (%)	Delta %	Preferred rule n (%)	Less restrictive rule n (%)	Delta %	Preferred rule n (%)	Less restrictive rule n (%)	Delta %	Preferred rule n (%)	Less restrictive rule n (%)	Delta %	Preferred rule n (%)	Less restrictive rule n (%)	Delta %
Pulses	6666 (97.97)	Not to be changed		136 (97.14)	Not to be changed		154 (100.00)	Not to be changed		154 (100.00)	Not to be changed		154 (100.00)	Not to be changed	
Milk and Yogurt	6670 (99.67)[2]	Not to be changed		140 (100.00)	Not to be changed		154 (100.00)	Not to be changed		154 (100.00)	Not to be changed		154 (100.00)	Not to be changed	
Cheese	6574 (98.24)[2]	Not to be changed		138 (98.57)	Not to be changed		152 (98.70)	Not to be changed		152 (98.70)	Not to be changed		152 (98.70)	Not to be changed	
Plant-based beverages and Yogurt[3]	112 (100.00)	Not to be changed													
Plant-based Cheese[3]	110 (98.21)	Not to be changed													
Fish or seafood	6769 (99.49)	Not to be changed		140 (100.00)	Not to be changed		154 (100.00)	Not to be changed		154 (100.00)	Not to be changed		154 (100.00)	Not to be changed	
All meat [1]	6692 (98.35)	Not to be changed		140 (100.00)	Not to be changed		154 (100.00)	Not to be changed		154 (100.00)	Not to be changed		154 (100.00)	Not to be changed	
Eggs	6721 (98.78)	Not to be changed		140 (100.00)	Not to be changed		154 (100.00)	Not to be changed		154 (100.00)	Not to be changed		154 (100.00)	Not to be changed	
Bread	6802 (99.97)	Not to be changed		140 (100.00)	Not to be changed		153 (99.35)	Not to be changed		154 (100.00)	Not to be changed		154 (100.00)	Not to be changed	
Tubers	5962 (87.62)	6795 (99.87)	12.24	120 (85.71)	140 (100.00)	14.29	152 (98.70)	154 (100.00)	1.30	154 (100.00)			154 (100.00)		
Rice	6517 (95.78)	6803 (99.99)	4.20	137 (97.86)	140 (100.00)	2.14	154 (100.00)			154 (100.00)			154 (100.00)		
Pasta	6796 (99.88)	Not to be changed		140 (100.00)	Not to be changed		154 (100.00)	Not to be changed		154 (100.00)	Not to be changed		154 (100.00)	Not to be changed	
Cereals	4775 (70.18)	6797 (99.90)	29.72	95 (67.86)	140 (100.00)	32.14	87 (56.49)	154 (100.00)	43.51	72 (46.75)	153 (99.35)	52.60	72 (46.75)	153 (99.35)	52.60
Fruit	6793 (99.84)	Not to be changed		140 (100.00)	Not to be changed		98 (63.64)	Not to be changed		93 (60.39)	Not to be changed		93 (60.39)	Not to be changed	
Vegetables	6804 (100.00)	Not to be changed		140 (100.00)	Not to be changed		146 (94.81)	Not to be changed		154 (100.00)	Not to be changed		154 (100.00)	Not to be changed	

**Red vegetables**	6713 (98.66)	6798 (99.91)	1.25	140 (100.00)			149 (96.75)	154 (100.00)	3.25	154 (100.00)			154 (100.00)		
**Green vegetables**	6601 (97.02)	6804 (100.00)	2.98	136 (97.14)	140 (100.00)	2.86	140 (90.91)	154 (100.00)	9.09	154 (100.00)			154 (100.00)		
**White vegetables**	6761 (99.37)	6804 (100.00)	0.63	140 (100.00)			135 (87.66)	152 (98.70)	11.04	152 (98.70)	154 (100.00)	1.30	152 (98.70)	154 (100.00)	1.30
**Yellow vegetables**	6779 (99.63)	6804 (100.00)	0.37	140 (100.00)			154 (100.00)			154 (100.00)			154 (100.00)		
**Purple vegetables**	6804 (100.00)			140 (100.00)			154 (100.00)			154 (100.00)			154 (100.00)		
**Multicolor vegetables**	6253 (91.90)	6789 (99.78)	7.88	128 (91.43)	140 (100.00)	8.57	152 (98.70)	154 (100.00)	1.30	154 (100.00)			154 (100.00)		

Data are presented as numbers of daily NPs (%). [1] All meat includes processed, red and white meat. [2] Data on milk and yogurt and on cheese does not include NPs for users with milk protein allergy in Spain (n=112 daily NPs), as the related food was not provided. The rules for all other food items were the same for both the regular menu and those for children with milk protein allergy. [3] The rules for dairy-free products refers just to NPs for users with milk protein allergy in Spain (n=112 daily NPs).

***Supplementary Table** S2. Compliance of weekly NPs in Spain and Türkiye with the daily rules from the CNR, categorized into those based on school menus, school cafeteria proposals or meals prepared at home.*

**COMPLIANCE OF WEEKLY MENUS WITH THE WEEEKLY RULES FROM THE CNR**															
	**Spain (n=992)**						**Türkiye (n=66)**								
	**NPs based on school menu (n=972)**			**NPs for meals prepared at home (n=20)**			**NPs based on school menu (n=22)**			**NPs based on school cafeteria (n=22)**			**NPs for meals prepared at home (n=22)**		
**Food items**	**Preferred rule n (%)**	**Less restrictive rule n (%)**	**Delta %**	**Preferred rule n (%)**	**Less restrictive rule n (%)**	**Delta %**	**Preferred rule n (%)**	**Less restrictive rule n (%)**	**Delta %**	**Preferred rule n (%)**	**Less restrictive rule n (%)**	**Delta %**	**Preferred rule n (%)**	**Less restrictive rule n (%)**	**Delta %**
**Pulses**	20 (2.06)	548 (56.38)	54.32	1 (5.00)	6 (30.00)	25.00	14 (63.64)	18 (81.82)	18.18	20 (90.91)	20 (90.91)	0.00	22 (100.00)		
**Chickpeas**	740 (76.13)	924 (95.06)	18.93	12 (60.00)	18 (90.00)	30.00	12 (54.55)	12 (54.55)	0.00	0 (0.00)	0 (0.00)	0.00	22 (100.00)		
**Lentils**	850 (87.45)	945 (97.22)	9.77	16 (80.00)	20 (100.00)	20.00	16 (72.73)	16 (72.73)	0.00	22 (100.00)	22 (100.00)	0.00	22 (100.00)		
**White/red beans**	388 (39.92)	948 (97.53)	57.61	5 (25.00)	18 (90.00)	65.00	0 (0.00)	10 (45.45)	45.45	0 (0.00)	22 (100.00)	100,00	0 (0.00)	22 (100.00)	100.00
**Other pulses**	109 (11.21)	917 (94.34)	83.13	3 (15.00)	18 (90.00)	75.00	0 (0.00)	0 (0.00)	0.00	0 (0.00)	0 (0.00)	0.00	0 (0.00)	0 (0.00)	0.00
**Milk and Yogurt**	852 (89.12)[1]	Not to be changed		16 (80.00)	Not to be changed		3 (13,64)	Not to be changed		8 (36,36)	Not to be changed		0 (0,00)	Not to be changed	
**Cheese**	22 (2.30)[1]	640 (66.95)[1]	64.64	1 (5.00)	12 (60.00)	55.00	12 (54.55)	22 (100.00)	45.45	2 (9.09)	2 (9.09)		0 (0.00)	22 (100.00)	100.00

**Plant-based beverages and Yogurt**[2]	15 (93.75)	Not to be changed													
**Plant-based Cheese**[2]	3 (18.75)	14 (87.50)	68.75												
**Fish or seafood**	302 (31.07)	972 (100.00)	68.93	6 (30.00)	20 (100.00)	70.00	22 (100.00)			22 (100.00)			22 (100.00)		
**Processed meat**	720 (74.07)	936 (96.30)[3]		13 (65.00)	20 (100.00)[3]		22 (100.00)	22 (100.00)[3]		22 (100.00)			22 (100.00)		
**Red meat**	869 (89.40)			15 (75.00)			19 (86.36)			22 (100.00)			22 (100.00)		
**White meat**	911 (93.72)	Not to be changed		20 (100.00)	Not to be changed		22 (100.00)	Not to be changed		22 (100.00)	Not to be changed		22 (100.00)	Not to be changed	
**Chicken**	938 (96.50)	Not to be changed		20 (100.00)	Not to be changed		22 (100.00)	Not to be changed		22 (100.00)	Not to be changed		22 (100.00)	Not to be changed	
**Turkey**	970 (99.79)	Not to be changed		20 (100.00)	Not to be changed		22 (100.00)	Not to be changed		22 (100.00)	Not to be changed		22 (100.00)	Not to be changed	
**Rabbit**	972 (100.00)	Not to be changed		20 (100.00)	Not to be changed		22 (100.00)	Not to be changed		22 (100.00)	Not to be changed		22 (100.00)	Not to be changed	
**Eggs**	798 (82.10)	Not to be changed		17 (85.00)	Not to be changed		22 (100.00)	Not to be changed		20 (90.91)	Not to be changed		22 (100.00)	Not to be changed	
**Carbohydrates**	965 (99.28)	Not to be changed		20 (100.00)	Not to be changed		20 (90.91)	Not to be changed		22 (100.00)	Not to be changed		22 (100.00)	Not to be changed	
**Bread**	972 (100.00)	Not to be changed		20 (100.00)	Not to be changed		22 (100.00)	Not to be changed		22 (100.00)	Not to be changed		22 (100.00)	Not to be changed	
**Tubers**	968 (99.59)	972 (100.00)	0.41	20 (100.00)			22 (100.00)			22 (100.00)			22 (100.00)		
**Rice**	972 (100.00)			20 (100.00)			22 (100.00)			22 (100.00)			22 (100.00)		
**Pasta**	972 (100.00)	Not to be changed		20 (100.00)	Not to be changed		22 (100.00)	Not to be changed		22 (100.00)	Not to be changed		22 (100.00)	Not to be changed	
**Cereals**	826 (84.98)	972 (100.00)	15.02	17 (85.00)	20 (100.00)	15.00	6 (27.27)	22 (100.00)		0 (0.00)	22 (100.00)	100.00	0 (0.00)	22 (100.00)	100.00
**Fruit**	546 (56.17)	Not to be changed		10 (50.00)	Not to be changed		1 (4.55)	Not to be changed		0 (0.00)	Not to be changed		0 (0.00)	Not to be changed	
**Vegetables**	421 (43.31)	965 (99.28)	55.97	6 (30.00)	20 (100.00)	70.00	5 (22.73)	22 (100.00)	77.27	20 (90.91)	22 (100.00)	9.09	0 (0.00)	22 (100.00)	100.00

Data are presented as numbers of weekly NPs (%). [1] Data on milk and yogurt and on cheese does not include NPs for users with milk protein allergy in Spain (n=16 weekly NPs), as the related food was not provided. The rules for all other food items were the same for both the regular menu and those for children with milk protein allergy. [2] The rules for dairy-free products refers just to NPs for users with milk protein allergy in Spain (n=16 weekly NPs) [3] The less restrictive rule applies to processed meat along with red meat.

Supplementary Figure S1. Site map of the SWITCHtoHEALTHY Family Application

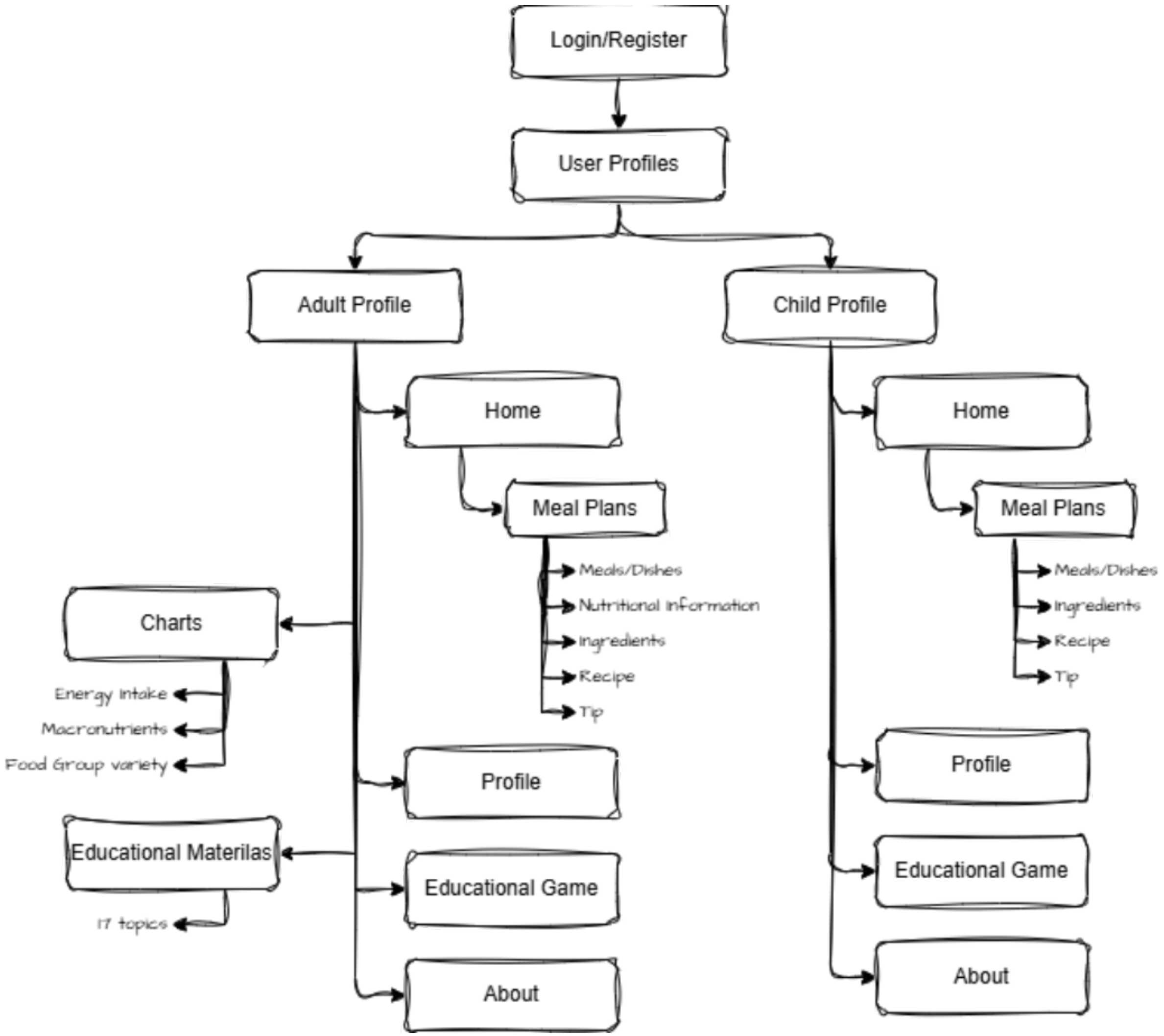

Supplement: Supplementary file 1 [file nutrients-17-03892-s001.zip › nutrients-4008702-supplementary.pdf]
